# Sex-specific outcomes of diabetic patients with acute myocardial infarction who have undergone percutaneous coronary intervention: a register linkage study

**DOI:** 10.1186/1475-2840-11-96

**Published:** 2012-08-11

**Authors:** Mai Blöndal, Tiia Ainla, Toomas Marandi, Aleksei Baburin, Jaan Eha

**Affiliations:** 1 Department of Cardiology, University of Tartu, 8 L. Puusepa Street, Tartu, 51014, Estonia; 2 Centre of Cardiology, North Estonia Medical Centre Foundation, 19 J. Sütiste Street, Tallinn, 13419, Estonia; 3 Quality Department, North Estonia Medical Centre Foundation, 19 J. Sütiste Street, Tallinn, 13419, Estonia; 4 Department of Epidemiology and Biostatistics, National Institute for Health Development, 42 Hiiu Street, Tallinn, 11619, Estonia; 5 Heart Clinic, Tartu University Hospital, 8 L. Puusepa Street, Tartu, 51014, Estonia

**Keywords:** Diabetes mellitus, Sex, Acute myocardial infarction, Percutaneous coronary intervention, Mortality, Outcome

## Abstract

**Background:**

The presence of diabetes mellitus poses a challenge in the treatment of patients with acute myocardial infarction (AMI). We aimed to evaluate the sex-specific outcomes of diabetic and non-diabetic patients with AMI who have undergone percutaneous coronary intervention (PCI).

**Methods:**

Data of the Estonian Myocardial Infarction Registry for years 2006–2009 were linked with the Health Insurance Fund database and the Population Registry. Hazard ratios (HRs) with the 95% confidence intervals (CIs) for the primary composite outcome (non-fatal AMI, revascularization, or death whichever occurred first) and for the secondary outcome (all cause mortality) were calculated comparing diabetic with non-diabetic patients by sex.

**Results:**

In the final study population (n = 1652), 14.6% of the men and 24.0% of the women had diabetes. Overall, the diabetics had higher rates of cardiovascular risk factors, co-morbidities, and 3–4 vessel disease among both men and women (p < 0.01). Among women, the diabetic patients were younger, they presented later and less often with typical symptoms of chest pain than the non-diabetics (p < 0.01). Women with diabetes received aspirin and reperfusion for ST-segment elevation AMI less often than those without diabetes (p < 0.01). During a follow-up of over two years, in multivariate analysis, diabetes was associated with worse outcomes only in women: the adjusted HR for the primary outcome 1.44 (95% CI 1.05 − 1.96) and for the secondary outcome 1.83 (95% CI 1.17 − 2.89). These results were largely driven by a high (12.0%) mortality during hospitalization of diabetic women.

**Conclusions:**

Diabetic women with AMI who have undergone PCI are a high-risk group warranting special attention in treatment strategies, especially during hospitalization. There is a need to improve the expertise to detect AMI earlier, decrease disparities in management, and find targeted PCI strategies with adjunctive antithrombotic regimes in women with diabetes.

## Background

Patients with acute myocardial infarction (AMI) have a worse short- and long-term prognosis than the general population [1,2]. During the last decades innovations in the treatment strategies, including the use of percutaneous coronary interventions (PCI) have considerably improved the prognosis [3-5]. However, women still have higher rates of complications compared with men, although the differences are often explained by their more adverse cardiovascular profile [6,7]. In particular, the presence of diabetes mellitus (DM) poses a challenge in the treatment of both sexes [8]. Previous studies have shown an interaction between sex and diabetes for congestive heart failure [9,10]. However, studies addressing the sex-specific impact of diabetes on the outcomes after AMI have included unselected cohorts where only a proportion of patients have undergone PCI [8,11,12]. Still, this may be the underlying cause for differences in sex-specific outcomes [13]. Furthermore, these studies have mostly concentrated on all-cause mortality only.

The aim of our study was to evaluate the sex-specific outcomes in terms of non-fatal AMI, revascularization, and all-cause mortality in diabetic and non-diabetic patients with AMI who have undergone PCI.

## Methods

We conducted a register linkage study by linking data from the following registries: the Estonian Myocardial Infarction Registry (EMIR), the Estonian Health Insurance Fund (EHIF) database, and the Estonian Population Registry (EPR). The study was approved by the Research Ethics Committee of the University of Tartu and by the Data Protection Inspectorate of Estonia.

The EMIR is a prospective registry including data on all consecutive patients hospitalized with the diagnosis of AMI [main diagnosis code I21 − I22 according to the International Statistical Classification of Diseases and Related Health Problems 10^th^ revision (ICD-10), [14]. The data collection in the EMIR complies with the Cardiology Audit and Registration Data Standards in Europe [15]. In the EMIR patients are classified as diabetic if they have a history of diabetes documented by a physician or the patients are diagnosed with diabetes during the current episode.

For the purpose of this study, we included consecutive AMI cases hospitalized into a tertiary care PCI centre, Tartu University Hospital, from 1 January 2006 until 31 December 2009. According to the internal audit conducted for the purpose of this study, the case coverage for the study period was 99.8%. We included only patients who underwent PCI during the index hospitalization. If a patient was admitted several times with AMI during the study period, we included only the first case of hospitalization into the study. EMIR provided the following data: (a) patient baseline characteristics (including diagnosis of diabetes), (b) prescription of medications during hospitalization and for outpatient use, (c) use of coronary angiography, revascularization, and echocardiography during the index hospitalization, (d) in-hospital outcomes.

Data on mortality during the follow-up were obtained from the EPR.

Data on the non-fatal AMI and repeated revascularization during follow-up were provided by the EHIF. In the Estonian health insurance system 95% of the 1.3 million inhabitants are insured. Consistency in reporting to the EHIF database and the validity of the data has been established [16]. Data on non-fatal AMI included the date of hospitalization and the diagnosis code according to the ICD-10 classification (I21 − I22, main diagnosis). The EHIF provided the data on the method and date of revascularization according to the Nordic Medico-Statistical Committee Classification of Surgical Procedures, version 1.6: percutaneous coronary intervention (procedure code FNG with numeric characters of the code) and coronary artery bypass graft (procedure codes FNA, FNC, and FNE with numeric characters of the codes) [17].

### Study outcomes

The primary composite outcome was defined as non-fatal AMI, repeated revascularization (coronary artery bypass-grafting; target or new lesion PCI), or all-cause mortality whichever occurred first. The follow-up started on the date of the PCI during the index hospitalization and ended if a case reached the primary outcome or the follow-up time ended (31 December 2010). We also studied all-cause mortality separately as a secondary outcome. For all-cause mortality the follow-up ended when the patient died or reached the date for the end of the follow-up.

### Statistical analysis

Categorical variables were expressed as percentages, and continuous variables as means and standard deviations (SDs), or as medians and interquartile ranges (IQRs). To compare the diabetic and non-diabetic patients by sex in respect to the baseline characteristics, procedural characteristics of PCI, prescription of medications, and outcomes the Chi-Square test for categorical variables and t-test for two independent samples or the Wilcoxon-Mann–Whitney test for continuous variables were used.

We used Cox’s proportional hazards regression to calculate hazard ratios (HRs) of primary and secondary outcome with the 95% confidence intervals (CIs) to compare outcomes of diabetic to non-diabetic patients by sex. In multivariate analysis the potential confounders from Table 1 were entered into the model if they were clinically relevant or showed univariable differences with a p < 0.05 between diabetic and non-diabetic patients of either gender. The assumption of proportional hazards assumed in the Cox proportional hazards model was assessed graphically. In our regression model we did not control for the recommendation of medical therapy because the timing of medication administration could not be determined from our methods of data abstraction, nor could we account for confounding by treatment indication given the study’s nonrandomized nature.

**Table 1:** Baseline characteristics of men and women with and without diabetes who have undergone percutaneous coronary intervention

	**Men**			**Women**		
	**Non-DM n = 905**	**DM n = 155**	**p**	**Non-DM n = 450**	**DM n = 142**	**p**
Mean age ± SD (years)	63.1 ± 12.0	65.0 ± 9.4	0.055	71.9 ± 9.9	69.3 ± 9.6	0.006
STEMI, %	66.9	63.2	0.378	70.2	62.0	0.066
Arterial hypertension, %	61.9	80.7	<0.001	76.4	91.6	<0.001
Dyslipidemia, %	64.3	62.5	0.679	72.7	69.7	0.495
Current smoking, %	44.2	30.3	0.001	14.2	7.0	0.024
Previous AMI, %	18.2	32.3	<0.001	15.8	25.4	0.010
Chronic heart failure, %	13.7	25.8	<0.001	12.4	29.6	<0.001
Previous stroke, %	5.4	9.0	0.078	5.3	7.8	0.288
Peripheral vascular disease, %	9.8	17.4	0.005	7.6	8.5	0.728
Previous PCI, %	8.7	20.7	<0.001	5.8	9.2	0.157
Previous CABG, %	4.3	3.9	0.802	2.4	3.4	0.025
Killip III − IV on admission, %	8.1	12.9	0.049	7.1	14.1	0.010
LVEF <40%, %	14.1	20.0	0.059	13.1	15.5	0.471
3 − 4 vessel disease, %	43.7	54.2	0.015	45.3	64.1	<0.001
Delay to FMC (hrs), %						
≤4	52.8	49.7	0.210	54.7	42.3	0.015
5–24	28.8	35.5		28.4	31.7	
>24	18.3	14.8		16.9	26.1	

AMI = acute myocardial infarction; CABG = coronary artery bypass graft surgery; DM = diabetes mellitus; FMC = first medical contact; LVEF = left ventricular ejection fraction; PCI = percutaneous coronary intervention; STEMI = ST-segment elevation myocardial infarction.

Two-sided p values < 0.05 were considered statistically significant. All analyses were performed using the Stata statistical software version 11 [18].

## Results

Of the 2330 patients with AMI hospitalized during the study period 1060 (74.7%) men and 592 (65.1%) women underwent PCI during the index episode (p < 0.001) and were included into the study. In the final study population 155 (14.6%) men and 142 (24.0%) women had diabetes (p *<* 0.001).

The baseline characteristics during the index hospitalization for men and women with and without diabetes are presented in Table 1. Overall, the patients with diabetes had higher rates of cardiovascular risk factors, co-morbidities, and 3–4 vessel disease among both men and women. Women with diabetes were younger and had longer delay times to first medical contact than those without diabetes. Among men 79.3% of non-diabetic and 77.4% of diabetic patients presented with typical symptoms of chest pain (p = 0.588). In women, those with diabetes presented less frequently with typical symptoms of chest pain than those without diabetes, respectively 68.3% and 79.3% (p = 0.007).

During the index hospitalization among women with ST-segment elevation myocardial infarction (STEMI) a significantly lower proportion of patients with diabetes than those without received reperfusion within 12 hours after symptom onset (Table 2). When assessing the utilization of medications during the index hospitalization, women with diabetes were found to receive aspirin less often than those without diabetes (Table 3). Men with diabetes received more often treatment with angiotensin-converting enzyme inhibitors and/or angiotensin II receptor blockers than men without diabetes. The rates of recommendation of the studied drugs for out-patient treatment were similar to the rates of drug utilization during the index hospitalization, though no differences within sex-groups were observed (data not presented).

**Table 2:** Procedural characteristics of percutaneous coronary intervention during the index hospitalization for men and women with and without diabetes

	**Men**			**Women**		
	**Non-DM %**	**DM %**	**p**	**Non-DM %**	**DM %**	**p**
STEMI	n = 605	n = 98		n = 316	n = 88	
Reperfusion within 12 hrs after symptom onset	72.1	67.4	0.337	75.0	60.2	0.006
Method of reperfusion*						
Fibrinolysis	15.6	18.2	0.593	11.0	11.3	0.941
Primary PCI	84.4	81.8		89.0	88.7	
NSTEMI	n = 300	n = 57		n = 134	n = 54	
Timing of PCI						
≤ 2 hrs after admission	18.0	7.0	0.039	17.2	14.8	0.694
≤ 12 hrs after admission	71.0	64.9	0.358	67.9	64.8	0.683

DM = diabetes mellitus; NSTEMI = non-ST-segment elevation myocardial infarction; PCI = percutaneous coronary intervention; STEMI = ST-segment elevation myocardial infarction.

*Among those who received reperfusion.

**Table 3:** Utilization of medications during the index hospitalization for men and women with and without diabetes who have undergone percutaneous coronary intervention

**Medications**	**Men**			**Women**		
	**Non-DM n = 905**	**DM n = 155**	**p**	**Non-DM n = 450**	**DMn = 142**	**p**
	**%**	**%**		**%**	**%**	
Aspirin	98.6	99.4	0.425	99.3	96.5	0.010
Clopidogrel	93.9	96.8	0.156	93.3	90.9	0.319
Heparin group	96.1	96.8	0.699	96.0	96.5	0.797
Glycoprotein IIb/IIIa receptor inhibitors	36.8	39.4	0.542	32.9	29.6	0.461
Beta-blockers	78.9	82.6	0.294	79.6	76.8	0.477
ACEI/ARB	79.6	87.7	0.017	86.7	84.5	0.516
Statins	75.9	76.8	0.816	75.8	73.9	0.658

ACEI = angiotensin-converting enzyme inhibitors; ARB = angiotensin II receptor blockers; DM = diabetes mellitus.

**Figure 1:**
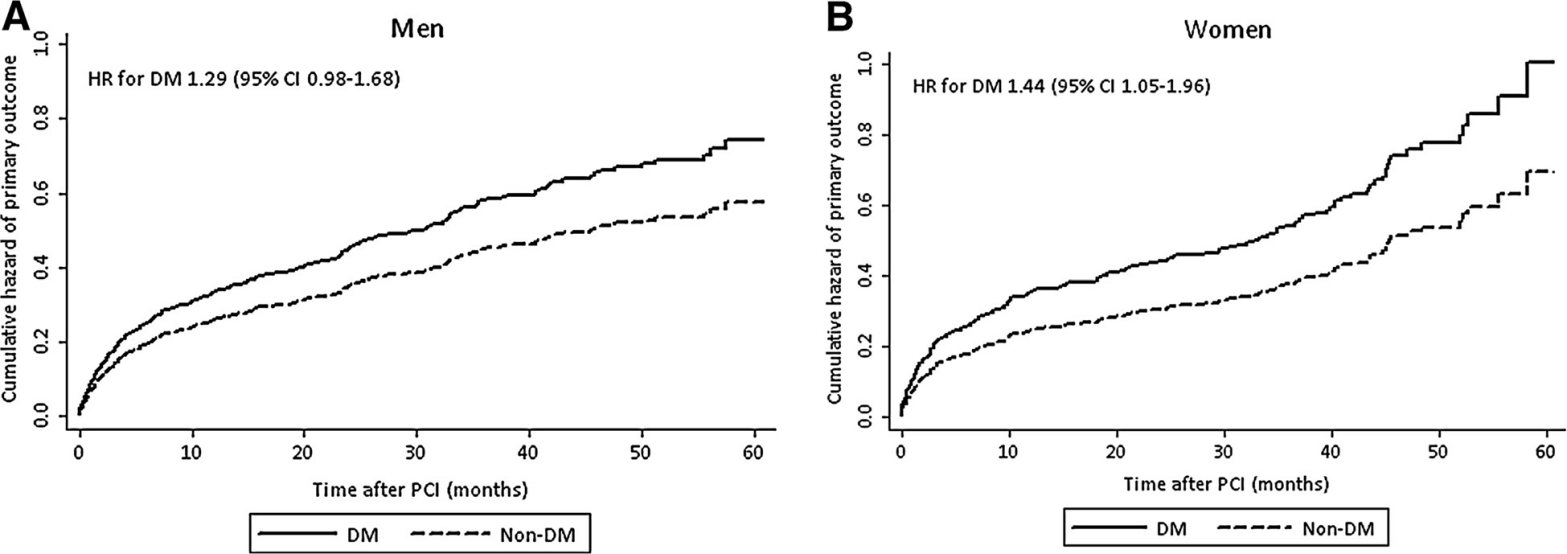
**Cumulative hazards of primary outcome (non-fatal AMI, repeated revascularization, or all-cause mortality whichever occurred first**) **in men and women with and without diabetes during follow-up after percutaneous coronary intervention.** CI = confidence interval; DM = diabetes mellitus group; HR = hazard ratio; PCI = percutaneous coronary intervention.

**Figure 2:**
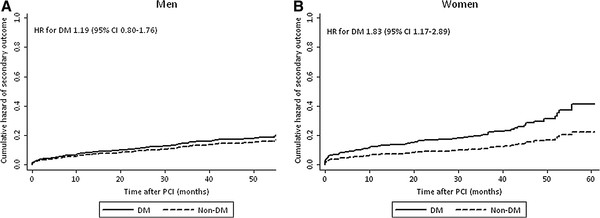
**Cumulative hazards of secondary outcome (all-cause mortality) in men and women with and without diabetes during follow-up after percutaneous coronary intervention.** CI = confidence interval; DM = diabetes mellitus group; HR = hazard ratio; PCI = percutaneous coronary intervention.

The median follow-up time for the primary outcome was 2.1 years (IQR 0.8 − 3.4) and for the secondary outcome 2.7 years (IQR 1.6 − 3.8) with no differences between the sex-groups. In univariate analysis, patients with diabetes both among men and women had a higher risk of primary and secondary outcome (Tables 4 and 5). In multivariate analysis, however, diabetes was associated with a higher risk of primary and secondary outcome only in women (Table 5). The Figures 1 and 2 demonstrate the cumulative hazards of the outcomes in diabetic and non-diabetic patients by sex.

**Table 4:** Outcomes during the follow-up among men and women with and without diabetes who have undergone percutaneous coronary intervention

	**Men**			**Women**		
	**Non-DM n = 905**	**DM n = 155**	**p**	**Non-DM n = 450**	**DM n = 142**	**p**
	**%**	**%**		**%**	**%**	
Primary outcome	37.6	49.7	0.004	36.0	52.1	0.001
Non-fatal AMI	9.9	12.3		7.3	12.0	
Revascularization	11.9	18.6		10.4	12.7	
Death	15.7	19.4		18.2	27.5	
Secondary outcome	18.2	23.8	0.099	18.7	29.6	0.006
Death during hospitalization	5.3	3.9	0.453	5.8	12.0	0.013

AMI = acute myocardial infarction; DM = diabetes mellitus.

**Table 5:** Crude and adjusted hazard ratios for outcomes during follow-up after percutaneous coronary intervention among men and women with diabetes compared to those without diabetes

**Outcome**	**Men**		**Women**	
	**HR (95% CI)**	**AHR (95% CI)**	**HR (95% CI)**	**AHR (95% CI)**
Primary^¥^	1.54 (1.20 − 1.98)*	1.29 (0.98 − 1.68)	1.60 (1.21 − 2.13)*	1.44 (1.05 − 1.96)*
Secondary^€^	1.53 (1.06 − 2.20)*	1.19 (0.80 − 1.76)	1.65 (1.11 − 2.44)*	1.83 (1.17 − 2.89)*

AHR = hazard ratio; adjusted for age, AMI subtype (STEMI or NSTEMI), arterial hypertension, dyslipidemia, current smoking, previous AMI, chronic heart failure, previous stroke, peripheral vascular disease, previous PCI, previous CABG, Killip class III − IV on admission, left ventricular ejection fraction <40%, and the number of diseased vessels (1–2 or 3–4 vessel disease), delay to first medical contact.

^¥^Primary outcome = non-fatal AMI, revascularization, or all-cause mortality.

^€^Secondary outcome = all-cause mortality.

*p *<* 0.05.

The in-hospital mortality of women with diabetes was three times higher than that of the men with diabetes, respectively 12.0% and 3.9% (p *=* 0.009). The overall rates of cardiogenic shock (4.5%), bleeding complications (4.4%), mechanical complications (4.4%), and stroke (0.7%) during the index hospitalization were low.

## Discussion

This register linkage study demonstrates that among patients with AMI who have undergone PCI, diabetes is associated with significantly worse outcomes in women than in men compared to those without diabetes. This excess risk seems to be largely driven by a high in-hospital mortality of women with diabetes.

There are few studies on this particular subject. Most of them have included mixed cohorts of patients with stable and unstable coronary artery disease undergoing PCI [8,11-13,19-21]. The results are in accordance with our findings and collectively suggest that the worse effect of diabetes on the outcomes in women might be related to the onset mechanisms of AMI, the procedural success of PCI as well as to the higher burden of cardiovascular disease (CVD) risk factors. Rosengren et al. [20] attributed much of the excess mortality in young women of less than 50 years of age to diabetes. The study of Champney et al. [21] demonstrated that the overall stronger effect of diabetes among women was more pronounced in the 1990ies than in the more recent years. These findings could be associated to the developments in the treatment strategies of high risk patients undergoing PCI.

In CVD risk management strategies diabetes may not only be a risk equivalent of a previous CVD but may actually confer a greater risk among women [22]. This could be explained by the fact that women and diabetics are more prone to develop diffuse small-vessel disease and therefore have more frequently diabetic cardiomyopathy. In addition, there are some fundamental biological differences in the composition of the atherosclerotic plaque between men and women [23-26]. So when a CVD event such as AMI occurs, women with diabetes have a worse prognosis due to a more serious underlying coronary artery disease and a more depressed myocardial function. Also the role of the lack of the ovarian hormones with cardiovascular protective effects in postmenopausal women is much discussed [27]. The mechanisms associated with the onset of AMI may be compromised to a greater extent in women than in men with diabetes as there are sex differences in the endothelial dysfunction, myocardial contractile function, neuroendocrinal regulatory mechanisms, sensitivity to aggregating stimuli of platelets, and tolerance to stress [27-30]. Conflicting results exist on the sex-specific association between admission hyperglycemia and the risk of in-hospital mortality in patients with AMI [31,32]. Still, the study of Kawamoto et al. [33] suggest that women have a more pronounced inflammatory reaction to hyperglycemia than men, making women with diabetes a high risk group for cardiovascular adverse events [34]. Furthermore, in women a higher rate of procedural complexity, peripheral complications, and bleeding after PCI has been reported [35].

There are several issues concerning the baseline characteristics that should be addressed when assessing the sex-specific outcomes of diabetic patients in the given study. Firstly, the female diabetic patients presented later and less often with typical symptoms of chest pain compared to the non-diabetics. Secondly, although the women with diabetes were younger, the rates of previous AMI and chronic heart failure were higher than in those without diabetes. As Norhammar et al. [36] demonstrate the poorer outcomes of younger women with diabetes compared to men are to a large extent related to an increased risk factor burden. Still, also the older women with diabetes seem to have a disadvantage compared to older men. The study of Leosdottir et al. [37] indicated that the older women with diabetes have a more pronounced risk factor clustering and worse self-rated health than older men. This adds to the excess cardiovascular risk of women with diabetes and in combination with the higher age could be the contributing factor for sex-specific outcomes of patients with diabetes. Therefore, in order to alleviate this unfavorable situation for women with diabetes, the improvements should target already the primary prevention strategies of AMI. The medical professionals as well as the patients with CVD should be more aware of the wider prevalence of atypical symptoms among women with and aim towards shorter pre-hospital delay times.

The study by Krämer et al. [38] suggests that the sex differences in the AMI mortality of patients with diabetes may be due to the fact that the diabetic men with CVD might be more thoroughly treated compared to the women. Interestingly, it has also been shown, that the control of hyperglycaemia and major CVD risk factors is less satisfactory in women than men [39]. In our study aspirin was recommended less frequently for women with diabetes than for those without. The analysis also showed that in women with STEMI, those with diabetes received less often reperfusion within 12 hours after symptom onset than those without diabetes. In the future research it is essential to identify the reasons for these disparities as they lead to larger infarct sizes, development of congestive heart failure, and decreased survival. Moreover, the success rate of primary PCI has been shown to be lower in women and in patients with diabetes [40,41].

When assessing the overall rates of outcomes it should be recognized that patients with AMI who have undergone PCI have a poor prognosis. With a median follow-up time of more than two years, almost half of the study population experienced a primary outcome event and by the end of the follow-up a fourth of the patients were dead. This emphasizes the need of cardiovascular risk adjusted lifestyle changes and proper use of guideline-recommended medications in the treatment of CVD and other co-morbidities such as diabetes in patients after AMI.

The strengths of our study are the use of an unselected real-life cohort of patients from a national register, the relatively long follow-up period, and the use of non-fatal AMI and revascularization as an outcome measure in addition to all-cause mortality.

We acknowledge the limitations of this study. Firstly, this is a register linkage study and there may be confounders that we were not able to adjust for, including consistence in the use of prescribed drugs after hospitalization. Secondly, we do not have detailed data on the type and duration of diabetes as well as the specific treatment and adequacy of glycaemic control in patients with diabetes during the index hospitalization and after hospital discharge. Thirdly, while suggestive of a sex difference in prognosis associated with diabetes, our epidemiological findings do not explain the possible pathological mechanisms underlying such a difference. Furthermore, the rates of non-fatal in-hospital complications are low and do not enable us to study the immediate causes of death in a systematic manner. Fourthly, the issue of selection bias arises as only a proportion of patients with an AMI, more frequently men, underwent PCI. Clinicians tend to refer younger patients with less co-morbidities for invasive procedures. Therefore the patients selected for PCI in our study should have a lower risk than those not selected; this applies both to men and women. However, as the women with AMI tend to be older and have a higher risk factor burden, the differences in the baseline characteristics between men and women persist. Nevertheless, this is an observational study reflecting management of patients with AMI in real life and we cannot exclude a possible selection bias. Still, in case of a selection bias the real mortality risks of the women selected for PCI, would be underestimated. Fifthly, as the differences in the outcomes are easier to be shown in a high-risk population than in low or intermediate-risk populations, the outcome differences among men could reach statistical significance in a larger study cohort. However, the number of men in the study was twice as high as that of the women. So in case worse outcomes for men with diabetes compared to those without could be demonstrated, the effect would be of low clinical importance.

## Conclusions

Diabetic women with AMI who have undergone PCI are a high-risk group warranting special attention in treatment strategies, especially during hospitalization. There is a need to improve the expertise to detect AMI earlier, decrease disparities in management, and find targeted PCI strategies with adjunctive antithrombotic regimes in women with diabetes.

## Abbreviations

ACEI, Angiotensin-converting enzyme inhibitors; AMI, Acute myocardial infarction; ARB, Angiotensin II receptor blockers; CABG, Coronary artery bypass graft surgery; CI, Confidence interval; CVD, Cardiovascular disease; DM, Diabetes mellitus; EHIF, Estonian Health Insurance Fund; EMIR, Estonian Myocardial Infarction Registry; EPR, Estonian Population Registry; FMC, First medical contact; HR, Hazard ratio; ICD-10, International Statistical Classification of Diseases and Related Health Problems 10^th^ revision; IQR, Interquartile range; LVEF, Left ventricular ejection fraction; NSTEMI, Non-ST-segment elevation myocardial infarction; PCI, Percutaneous coronary intervention; SD, Standard deviation; STEMI, ST-segment elevation myocardial infarction; TIMI, Thrombolysis in Myocardial Infarction.

## Competing interests

The authors declare that they have no competing interests.

## Authors’ contributions

MB, TA, TM, AB, and JE participated in the design of the study and in writing the manuscript. MB and AB performed the statistical analysis. All authors read and approved the final manuscript.

## Authors’ information

MB, TA, TM, and JE are the members of the working group of Acute Coronary Syndromes of the Estonian Society of Cardiology as well as the members of the Scientific Board of the Estonian Myocardial Infarction Registry.
